# Effects of date palm pollen (Phoenix dactylifera L.) and Astragalus ovinus on sperm parameters and sex hormones in adult male rats

**Published:** 2014-10

**Authors:** Fouad Mehraban, Mehrzad Jafari, Mehdi Akbartabar Toori, Hossein Sadeghi, Behzad Joodi, Mostafa Mostafazade, Heibatollah Sadeghi

**Affiliations:** 1 *Medicinal Plant Research Center, Yasuj University of Medical Sciences, Yasuj, Iran*; 2 *Student Research Committee, Yasuj University of Medical Sciences, Yasuj, Iran.*; 3 *Cellular and Molecular Research Center, Faculty of Medicine, Yasuj University of Medical Sciences, Yasuj, Iran.*; 4 *Social Determinants of Health Research Center, Yasuj University of Medical Sciences, Yasuj, Iran *; 5 *Department of Pharmacology, Faculty of Medicine, Yasuj University of Medical Sciences, Yasuj, Iran.*

**Keywords:** *AstragalusOvinus*, *Datepalmpollen*, *Infertility*, *Rats*, *Gonadalhormones*

## Abstract

**Background:** Date Palm Pollen *(DPP)* and *Astragalus genus* are used in some countries for the treatment of infertility.

**Objective:** This study was designed to investigate effects of DPP and *Astragalus ovinus* (*A.Ovinus*) on fertility in healthy adult male rats.

**Materials and Methods: **Thirty-six rats were divided into six groups (n=6) including control and five treatment groups. DPP (120, 240 and 360 mg/kg) and *A.ovinus* (100, 500 mg/ kg) were orally given to the treatment groups. After thirty-five days, blood samples were taken to determine serum levels of FSH, LH, testosterone and estradiol. Weight of testis and epididymis, sperm count, sperm motility, seminiferous tubules diameter (STD), germinal cell layer thickness (GCLT), sertoli, leydig and spermatogonia cells were also evaluated.

**Results: **DPP at the of 120 and 240 mg/kg doses significantly raised the ratio of testis or epididymis to body weight, sperm count, sperm motility , and estradiol level compared to the control group (p<0.05). LH and testosterone levels only noticeably increased at 120 mg/kg of DPP (p<0.01 and p<0.001 respectively). STD increased in the three applied doses (p=0.001). *A. ovinus* extract at the indicated doses produced a significant reduction in the ratio of testis or epididymis to body weight and sperm motility (p<0.05). Sperm count, spermatogonia, leydig cells and FSH level decreased at dose of 500 mg/kg. Furthermore, GCLT, spermatogonia cells, and serum estradiol level increased at 100 mg/kg dose of *A. ovinus*.

**Conclusion:** Our findings indicate that DPP could improve fertility factors, while *A.ovinus* can exhibit deleterious effects on gonad and sperm parameters in rats.

## Introduction

Infertility is a complex disorder with medical and psychosocial important that defined as one year of regular and unprotected intercourse without conception (1). Fifteen percent of married couples are affected by infertility that about 20% of them is attributed to the male. About 50% of male infertility is treatable (2, 3). Incomplete development of testis, diseases of reproductive system, increase scrotal temperature, immunological problems, endocrine disturbances, lifestyle and environmental factors and nutritional factors have been considered as the main causes of male infertility (4-6). 

To present many agents have been proposed to treat male infertility particularly plants or their derivatives have a long folklore of use in the enhancing of libido and fertility. For example *Date Palm Pollen *(*DPP*) and some genus of *Astragalus* has been used for many years in folk medicine in Middle East and some Asian countries for promotion of good health and improving male or female fertility (7-10). Pharmacological effects of a number of these plants such as *DPP*, *Astragalus* *membranaceus*, *Asparagus racemous* and *Withania somniferous* on fertility have been evaluated *in vitro *and *in vivo *conditions (11-12). 

Recently, some animal studies have been reported that *DPP* and* Astragalus membranaceus* can ameliorate the deleterious effects of cadmium and cyclophosphamide on reproductive toxicity (7-8). Furthermore, phytochemical studies have been confirmed presence of estrone, α-amirin, triterpenoidal saponins and flavonoids in *DPP* (12, 14, 15). DPP extracts also contain cholesterol, rutin, carotenoids, oestrones- gonad stimulating components that can improve male infertility and elicit gonadotrophin activity (12, 16).

*Astragalus* is a large genus, belonging to the legume family *Fabaceae* and the subfamily *Faboideae* (17). Three important kinds of pharmacologically active components including polysaccharides, saponins and phenolics and some toxic compounds such as the indolizidine alkaloids, aliphatic nitro compounds and the seleniferous derivatives have been identified in Astragalus genus (18). Therefore the aim of present study was to evaluate the effects of *DPP* and specie of astragalus (*Astragalus* ovinus) on sperm characteristics, testicular histomorphometric and serum concentration of hormones in more details in rats. 

## Materials and methods

**Plant materials**

*DPP* and the aerial parts of* Astragalus ovinus (A.ovinus)* were collected from Fars Province (Iran) and the suburbs of Yasuj (Iran) at the end of spring 2012, respectively. The plants identified by Dr. A. Jafari (Department of Botany, Center for Research in Natural Resource and Animal Husbandry, Yasuj University, Yasuj, Iran).

**Preparation of DPP suspension and hydro-alcoholic extract** ***A.ovinus***

The pollens were separated from the kernels with a fine gauze sieve and kept refrigerated (4^o^C). The aqueous suspension was freshly prepared daily by adding distilled water to powdered pollen with stirring for 10 minutes on a magnetic stirrer till complete dispersion (12). 

The aerial parts of *A.ovinus* were air-dried, protected from direct sunlight, and subsequently powdered. Two hundred grams of the dry powder was extracted in a 48 hour period with 1 L of mixture of ethanol: water (70:30 ratios) at 48^o^C. The solvent was completely removed by rotary vacuum evaporator at 60^o^C and finally was dried (this output extract was 25%). The resulting sample was powdered and sealed for subsequent use.

**Animals and experimental procedures**

At this experimental study, thirty-six healthy adult male Sprague-Dawley rats weighing 200 to 250 grams were selected for the present study. They were obtained from the animal house of Yasuj University of Medical Sciences. They housed under controlled conditioning (22±1^o^C constant temperature, 55% relative humidity, 12 h lighting cycle), and kept under laboratory conditions two weeks prior to the experiment for acclimatization and received standard diet and water ad libitum during the study period.

After the adaptation period, the rats were randomly divided into six groups of 6 rats each: the first group of rats (control) only received distilled water orally. The second, third and fourth groups of animals were given one ml of suspension of *DPP* in distilled water containing 120, 240, 360 mg/kg body weight by gavages 5^th^ and 6^th^ groups also were orally administered with one ml each corresponding to 100, 500 mg/kg body weight of the plant extract of *A.ovinus*, respectively (8, 16, 18). All treatments were maintained daily for thirty-five consecutive days.

For the extract to have effect, the rats need a period of 48-52 days for the exact spermatogenic cycle (20). More than half the period is necessary to determine the effects of *DPP* and *A.ovinus* on fertility potential of male rats (12, 16). The study was approved by the ethic committee of Yasuj University of Medical Sciences. 

**Collection of blood, reproductive organs and tissue samples**

At the end of the experimental period, the rats were individually weighed and anesthetized with diethyl ether and sacrificed. Blood samples were collected from the heart. Blood serum was obtained by centrifugation at 3000 rpm for 20 minutes, and serum stored at -80^o^C for measurement of hormones (12). Afterward, all testes, epididymides and seminal vesicles were excised and weighed, along with the relative weight of organs was calculated as mg/g body weight (relevant data are not shown). 

The all testicular tissues were fixed in 10% formalin, and then embedded in paraffin and all tissue blocks sectioned at serial sections of 5 µm and were stained with haematoxylin and eosin (H&E) for histopathologic examination (21). Subsequently, histomorphometric parameters were measured with Olympus IX71 microscope and Olysia software to measure seminiferous tubules diameter (STD), germinal cell layer thickness(GCLT) sertoli, leydig and spermatogonia cells means per unit area (22).

**Measurement of hormones**

Serum concentrations of testosterone and estradiol were measured with using micro plate enzyme immunoassay (ELISA) and expressed as ng/ml and nmol/L respectively, follicle stimulating hormone (FSH), leuteinizing hormone (LH) were assayed based on the Micro Plate Immunoenzymometric assay (ELISA) as described in the instructions provided by manufacturer’s kits and expressed as mIU/mL. All of animal kits purchased from Monobind Inc; USA.

**Epididymal sperm counts and motility**

The scrotal sacs of rats were opened, the testes removed, trimmed of fat; and the cauda epididymides were removed for sperm analysis. The slides on which the sperm cells were counted were heated to 37^o^C until the time of the analysis. The analysis was carried out at room temperature using one epididymis of each rat. The percentage of sperm motility was calculated using the number of live sperm cells over the total number of sperm cells, from two samples from one epididymis of each rat. Sperm count was achieved using the new improved Neubauer's counting chamber. The Neubauer chamber is a thick crystal slide with the size of a glass slide. (30×70 mm and 4 mm thickness).

The epididymal fluid was diluted with normal saline by adding 0.9 ml to 0.1 of crushed epididymis. The counting chamber was next charged with a cover slip until a rainbow picture was seen at the edges. This chamber was afterward filled with sperm fluid and placed under a binocular light microscope using an adjustable light source. The ruled part was then focused and the number of spermatozoa counted in five 16-celled squares. The sperm concentration was subsequently calculated and multiplied by 10^6^ and expressed as X ×10^6^/ml, where X is the number of sperm in a 16- celled square. In order to investigate morphologically abnormal sperms, the slides were stained with eosin-nigrosin and then examined under a microscope with a magnification of 400. 300 sperm per slide were analyzed (2100 cells per group) and abnormal total were expressed as percentage (23).

**Statistical analysis**

All quantitative data derived from this study were expressed as mean±SEM. The statistical differences between groups were assessed by one-way ANOVA and LSD post hoc test using the SPSS software package for Windows. P<0.05 was considered to be statistically significant. 

## Results

**Epididymal sperm characteristics**

Epididymal sperm characteristics are shown in table I. There was a significant increase with administration of 120 and 240 mg/kg doses of *DPP* in sperm motility: motile (p<0.001), sperm count (p<0.01), and significant decrease was observed in immotile sperm (p=0.032), total abnormality of sperm (p<0.01) in dose of 120 mg/kg as compared with the control group. There were no significant differences in the 360 mg/kg group from *DPP* in terms of these parameters when compared with the control group. Marked reductions in both *A.ovinus* groups were observed in sperm motility: motile (p<0.01), sluggish (p<0.001) and marked augment in immotile sperm (p=0.022) when compared with the control group. *A.ovinus* extract at dose of 500 mg/kg caused a significant reduction (p=0.041) in sperm count while it non-significantly decreased at 100 mg/kg dose. There were no significant differences among the groups from *A.ovinus* regarding total abnormality of sperm compared with the control group.

**Testicular histopathology and histomorfometric study**

**Figure 1:**
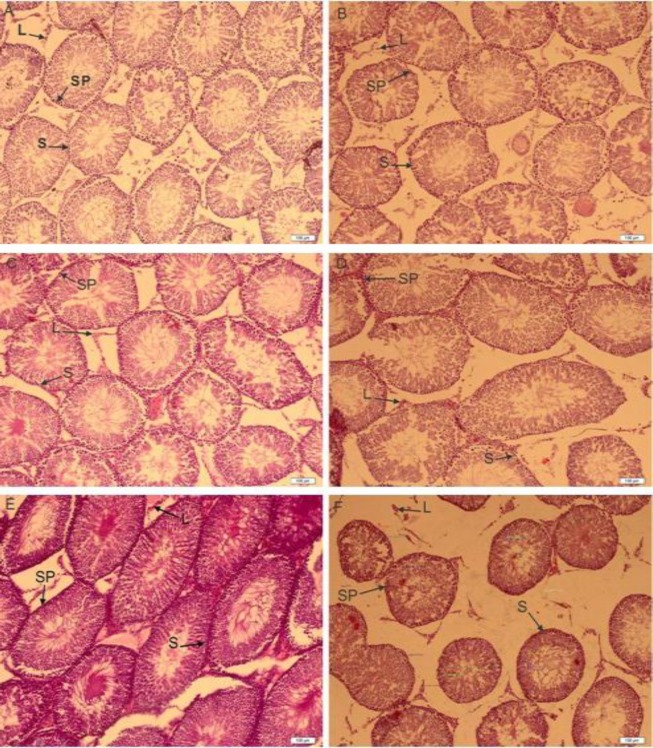
The effects of thirty-five days of treatment with the *DPP* suspension in distilled water and hydro alcoholic extract of *A. ovinus *on rat testis histology. Control (A), 120, 240 and 360 mg/kg of *DPP* treated groups (B, C and D respectively), 100 (E) and 500 mg/kg (F) of *A.ovinus* treated groups. Treatment of rats with various doses of *DPP*, STD increased significantly and there was an insignificant increase in GCLT and spermatogonia cells. There were no differences in administration of 360 mg/kg of *DPP *(D). At E figure marked increased was obvious in the GCLT and spermatogonia cells whereas F figure shown decreased the number of leydig and spermatogonia cells. (Abbreviations used in Figure 1: SP: Spermatogonia cells, S: Sertoli cells, L: Leydig cells).

Histological appearances of testicular tissues of control and treated groups with *DPP* and *A.ovinus* are shown in figure 1. The histopathological changes such as STD, GCLT of the testis, number of sertoli, leydig and spermatogonia cells are shown in table II. After treatment of rats with various doses of *DPP*, STD increased significantly (p<0.01) and there was an insignificant increase in GCLT and spermatogonia cells. 

At both doses of *A.ovinus* no significant differences were observed in STD, but a marked increase was obvious in the GCLT and spermatogonia cells (p<0.01) with 100 mg/kg of *A.ovinus* whereas dose of 500 mg/kg *A.ovinus* decreased the number of leydig (p=0.004) and spermatogonia cells (p=0.013). There were no statistically significant differences among any of the groups in terms of sertoli cells (Table II).

**FSH, LH, testosterone and estradiol hormones**

**Figure 2:**
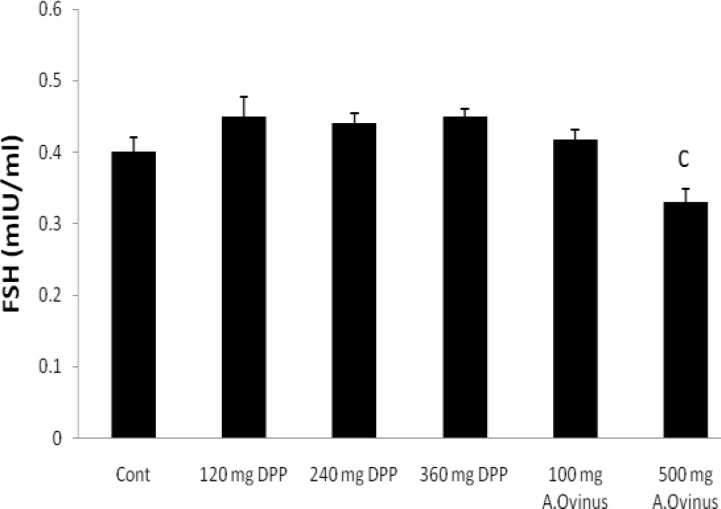
Effects of *DPP* and *A.Ovinus* on serum FSH levels in six groups (n=6). Values are mean±SEM, c: p<0.05 vs. Control group using one-way ANOVA and LSD post hoc test.

**Figure 3:**
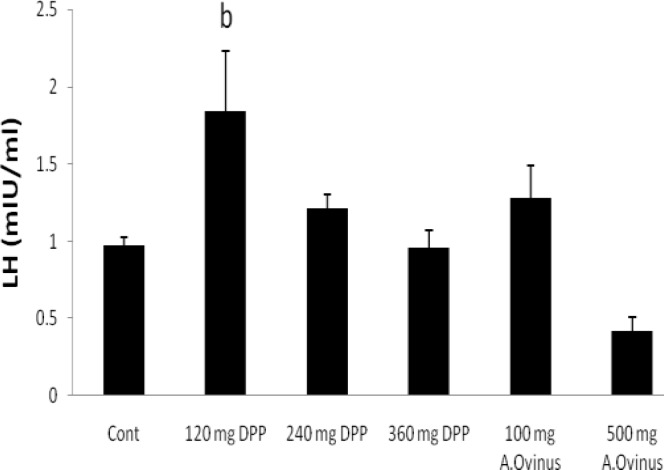
Effects of *DPP* and *A.ovinus* on serum LH levels in six groups (n= 6). Values are mean±SEM, b: p<0.01 vs. Control group using one-way ANOVA and LSD post hoc test.

**Figure 4:**
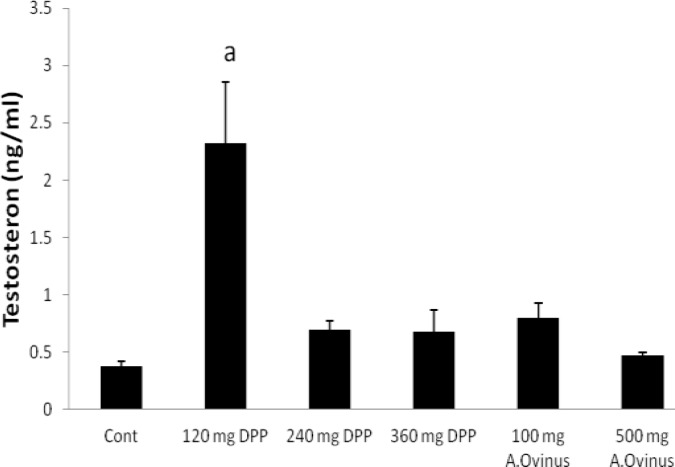
Effects of *DPP* and *A.ovinus* on serum testosterone levels in animal groups (n= 6). Values are mean±SEM, a: p<0.001 vs. Control group using one-way ANOVA and LSD post hoc test.

**Figure 5:**
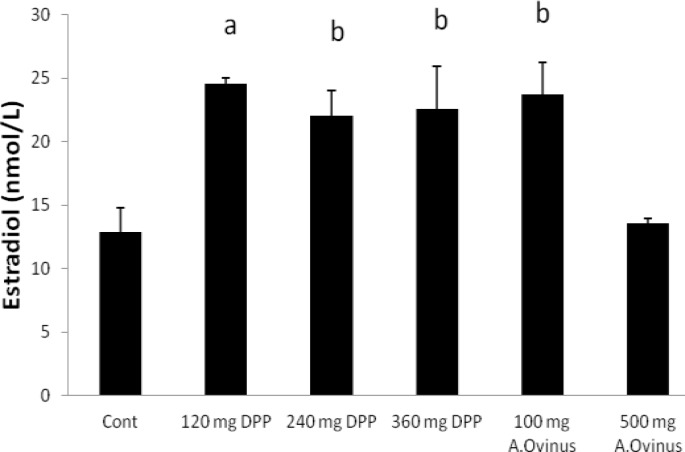
Effects of *DPP* and *A.ovinus* on serum estradiol levels in animal groups (n= 6). Values are mean±SEM, a: p<0.001, b: p<0.01, vs. Control group using one-way ANOVA and LSD post hoc test.

There were no significant differences among any of the groups regarding FSH as compared to the control group, excepting dose of 500 mg from *A.Ovinus* which reduced level of serum FSH (p=0.013) (Figure 2). Although dose of 120 mg/kg *DPP *increased LH level (p<0.01) but other groups was not statistically significant when compared to the control (Figure 3). Serum testosterone level elevated only at dose of 120 mg/kg of *DPP* significantly (p<0.001) in comparison to control group (Figure 4). All the doses of *DPP* and *A.ovinus* at 100 mg/kg produced significant increased (p=0.000 in 120 mg/kg DPP and p<0.003 in rest) estradiol levels (Figure 5).

**Table I:** Effects of *DPP* and *A. ovinus* on epididymal sperm characteristics in rats

**Parameters**	**Sperm motility (%)**				
**Groups**					
	**Motile**	**Sluggish**	**Immotile**	**Abnormal sperm rate (%)**	**Sperm count (million ⁄ml)**
Control	23.7 ± 1.34^a^	33.6 ± 0.89^a^	42.3 ± 2.28^a^	4.02 ± 0.02^a^	193.6 ± 14.42^a^
120 mg *DPP*	43.2 ± 2.14^b^	33.5 ± 0	23.2 ± 1.67^d^	3.3 ± 0.2^c^	376.4 ± 9.23^b^
240 mg *DPP*	38.7 ± 1.2^b^	33.2 ± 1.85	28.1 ± 1.56	4.02 ± 0.02	287.9 ± 16.29^c^
360 mg *DPP*	25.6 ± 0.88	37.2 ± 2.41	36.4 ± 2.54	3.6 ± 0.06	198.84 ± 11.85
100 mg *A. Ovinus *1	18 ± 1.97^c^	18.3 ± 2.24^b^	62.9 ± 2.65^d^	3.8 ± 0.25	154.2 ± 17.03
500 mg *A. Ovinus*	14.1 ± 0.86^b^	20.6 ± 2.21^b^	64 ± 2.68^d^	3.9 ± 0.19	136.4 ± 8.24^d^

The mean differences between the values bearing different superscript letters within the same column with control group are statistically significant using one-way ANOVA and LSD post hoc test

a, b : p<0.001

a, c : p<0.01

a, d : p<0.05

**Table II:** Effects of *DPP* and *A. ovinus* on histopathological and histomorfometric parameters of testis in rats

**Groups**	**Parameters**				
	**STD (µm)**	**GCLT (µm)**	**Sertoli cells**	**leydig cells**	**spermatogonia cells**
Control	274.23 ± 4.07^a^	71.23 ± 5.62^ a^	1.37 ± 0.18	7.87 ± 0.29^a^	70.12 ± 3.29^a^
120 mg DPP	300.02 ± 9.18^c^	83.05 ± 2.77	1.48 ± 0.18	7 ± 0.46	78.25 ± 2.86
240 mg DPP	300.14 ± 5.46^c^	82.88 ± 3.82	1.5 ± 0.19	6.87 ± 0.44	76.25 ± 6.26
360 mg DPP	300.16 ±5.54^c^	82.34 ± 3.84	1.55 ± 0.16	6.88 ± 0.23	75.73 ± 4.72
100 mg A. o	272.92 ± 4.65	89.40 ± 7.18^c^	1.5 ± 0.19	7.5 ± 0.37	87.37 ± 2.75^c^
500 mg A. o	272.14 ± 4.66	73.17 ± 5.38	1.37 ± 0.18	6.37 ± 0.42^c^	54.62 ± 2.55^d^

Data were presented as Mean±SEM. The mean differences between the values bearing different superscript letters within the same column with control group are statistically significant using one-way ANOVA and LSD post hoc test

a, b : p<0.001

a, c : p<0.01

a, d : p<0.05

## Discussion

Globally, infertility affects about 50-80 million couples at some point of their reproductive lives with a variety of biological and behavioral determinants. Various medicinal plants ranging from *DPP* and *Astragalus* genus have been implicated in male infertility (8-10, 13, 18, 24). Fortunately, quite a few countries in the world are gifted with plant biodiversity, and there is currently an emanating awareness about the significance of plant remedies in the health care delivery system. In many parts of the world, efforts are now being aimed at investigating therapeutic efficacy of locally available medicinal herbal plants. The beneficial role of medicinal plants in the treatment of male infertility has been indicated numerously (25).

The present results indicate that *DPP* with increasing dose decreased the epididymal sperm count and motility and moreover augmented the percentage of sperm abnormalities. These effects were associated with an enhancement in STD at all of doses. Similar to our results Turk *et al*, Bin-seng *et al* have also shown that pomegranate juice and fractions of quassinoid-rich *Eurycoma longifolia* extract consumption significantly increased epididymal sperm concentration and motility, spermatogenic cell density, STD and GCLT and decreased abnormal sperm rate when compared to that of the control (6, 26). In this investigation, it was found that treating male rats with the hydro-alcoholic extract of *A.ovinus*, at two doses, decreased significantly epididymal sperm motility. 

GCLT and spermatogonia cells showed increase at 100 mg/kg dose whereas sperm count, spermatogonia and leydig cells showed reduction at the dose of 500 mg/kg. The results show that *A.ovinus* extract in group F (Figure 1) causes increased tissue fluid, on the other hand, distance between the semeniferous tubes and an increase of congestion in blood vessels and reduced blood circulation as a result. When the structure of testis from the quality point of view was histopathologically examined; degeneration, necrosis and interstitial edema were detected in testis of F group when compared to the others group. Desquamative germinal cells and the deceleration of spermatogenesis were seen in the lumen of certain semeniferous tube of F group. Low epididymal sperm concentration may correlate with suppressed concentration of testosterone in the circulation and destruction of the wall of seminiferous tubules (ST) and consequently low fertility (27, 28). 

Also FSH will bind to the sertoli cells to facilitate spermatogenesis in ST (29). Reducing of sperm count at *A.ovinus* with 500 mg/kg dose might be due to the significant reduction in serum FSH concentration. Chronic poisoning of *Astragalus *species that are able to accumulate selenium, may reduce in reproductive efficiency   (30) . The result of the present investigation is consistent with the mentioned studies. Whereas the results of the Kim *et al* showed that the *Astragalus membranaceus* treatment increased diminished sperm count and motility in mice treated with cyclophosphamide (8). Increase in LH concentration observed in the treated-rats with 120 mg/kg of *DPP* will confer the increase in testosterone concentration which is the case in this the present study. This may be an indication that the *DPP* has stimulatory effect on the hypothalamic-pituitary axis of the male rats (31). 

The observed improvement potency of *DPP* may be due to presence of steroids, flavonoids, saponins, and lipid which may increase sexual behavior and have a positive effect on the sperm quality (12, 19). In other words, they stimulate the endogenous testosterone levels possibly by raising the level of LH (31). In the present study administration of *DPP* suspension significantly increased the testosterone and LH concentrations without marked augment of FSH when compared to that of the control group (Figure 2, 3, 4). Similar to our result, Pollen powder that was packed as 500 mg in capsule for treatment of twenty-five infertile men significantly increased the FSH serum, in addition to LH and testosterone levels (4). 

Abedi *et al* suggested that maximum effect for estradiol and testosterone was observed in rats treated with dose 140 mg/kg of *DPP *which led to maximum effect for sexual behavior of male rats which is close to our effective dose (120 mg/kg) (19). In another study by Bawazir Talbina (barley water) increased the plasma levels of testosterone that had a positive correlation with increased active spermatogenesis and significant rise of number of mature sperms (32).

Alkaloids of *DPP* also have estrogenic properties (19). It is also known that the estradiol components of *DPP* play an effective role in regulating the renewal of spermatogenic cells and male reproductive tissues that possess oestrogen receptors (12). In the present study, *DPP *at all of doses significantly augmented estradiol levels. Moreover, rats treated with *A.ovinus* increased serum estradiol level at dose of 100 mg/kg and lack of increase in estradiol at 500 mg/kg dose is due to the destructive effects on leydig and spermatogonia cells as estrogen is synthesized in male reproductive system by at least three different cell types, sertoli, leydig and germ cells (33). 

The increase in the testes- or epididymis-body weight ratio (results are not shown) observed following the administration of the *DPP* especially at doses of 120 mg/kg and 240 mg/kg may be attributed to increased secretory activity of the testes which in the present study is supported by the increase in the concentrations of testosterone and estradiol (Figure 4, 5) (34). Our result was similar to Yakubu and Afolayan’s study that weights of testes were enhanced as a result of consumption of Bulbine natalensis Baker stem extract at doses of 25 and 50 mg/ kg (35). The decline was observed in the testes- or epididymis-body weight ratio (Results are not shown) following the treatment of the *A.ovinus* extract at two doses may be attributed to cellular constriction and may as a result of presence of aliphatic nitro compounds or swainsonine, an indolizidine alkaloid or selenium accumulation that these compounds are responsible for the toxicity (34, 36).

## Conclusion

Based on the results of the present study, we conclude that the suspension of *DPP* in distilled water may act as a fertility agent. This is demonstrated by the increase in the fertility parameters (sperm count and motility, LH, testosterone and estradiol levels, diameter of seminiferous tubules) in the treated rats. Hydro alcoholic extract of *A.ovinus* may act against it, as an antifertility agent, especially in high doses. In addition, histological results of the testes of the treated rats showed disintegration of the seminiferous tubule walls and arrest in spermatogenesis.

## Footnote

***This article extracted from M.Sc. thesis. (Fouad Mehraban)***
